# Cardiac Arrest During the Medical Management of Left Ventricular Outflow Tract Obstruction Following the Transcatheter Aortic Valve Implantation

**DOI:** 10.7759/cureus.55026

**Published:** 2024-02-27

**Authors:** Yuka Saika, Ryo Wakabayashi, Hiroki Ichiyanagi, Aki Suzuki, Nobukazu Sato

**Affiliations:** 1 Clinical Training Center, Tokyo Saiseikai Central Hospital, Tokyo, JPN; 2 Department of Anesthesiology, Tokyo Saiseikai Central Hospital, Tokyo, JPN

**Keywords:** cardiac arrest, left ventricular outflow tract obstruction, negative inotropes, systolic anterior motion, transcatheter aortic valve implantation

## Abstract

Systolic anterior motion of the mitral valve and left ventricular outflow tract obstruction are complications following transcatheter aortic valve implantation and can lead to hemodynamic collapse. Medical management for those complications is usually centered on a reduction in left ventricular contractility with negative inotropes. An 88-year-old woman underwent transcatheter aortic valve implantation for severe aortic stenosis. Hemodynamic collapse and exacerbation of mitral regurgitation occurred immediately after valve implantation. For suspected left ventricular outflow tract obstruction, medical management centered on negative inotropes was performed. Hemodynamics and left ventricular outflow tract obstruction improved over time; however, the oxygen supply-demand imbalance progressed. On postoperative day 5, the patient suddenly went into pulseless electrical activity. Cardiopulmonary resuscitation was performed for three minutes, resulting in the return of spontaneous circulation. Subsequent refractory hypotension and oxygen supply-demand imbalance improved with continuous infusion of adrenaline, dobutamine, and phenylephrine. Her hemodynamics remained stable after she was weaned off the pressor infusions, and negative inotropes were not required again. In summary, the cause of cardiac arrest was possibly due to excessive negative inotropic effects even though the effects contributed to improvement of left ventricular outflow tract obstruction. Anesthesiologists and intensivists should recognize the risk of cardiac arrest induced by negative inotropic effects and use negative inotropes with rigorous hemodynamic monitoring, even when left ventricular outflow tract obstruction is treated effectively.

## Introduction

Systolic anterior motion of the mitral valve (SAM) and left ventricular outflow tract (LVOT) obstruction are complications following transcatheter aortic valve implantation (TAVI) and can lead to hemodynamic collapse [1]. As the mechanism by which these complications occur, it has been suggested that the sudden decrease of LV pressure after TAVI reduces the area of the LVOT and increases flow velocity across the LVOT [2]. From this pathophysiological perspective, medical management for these complications is usually centered on a reduction in LV contractility with negative inotropes, an increase of LV afterload with vasoconstrictors, and an increase of LV preload by fluid infusion [1-3]. Here, we report a case of cardiac arrest despite effective medical management for SAM and LVOT obstruction following TAVI. Written informed consent for publication of this case report was obtained from the patient.

## Case presentation

**Figure 1:**
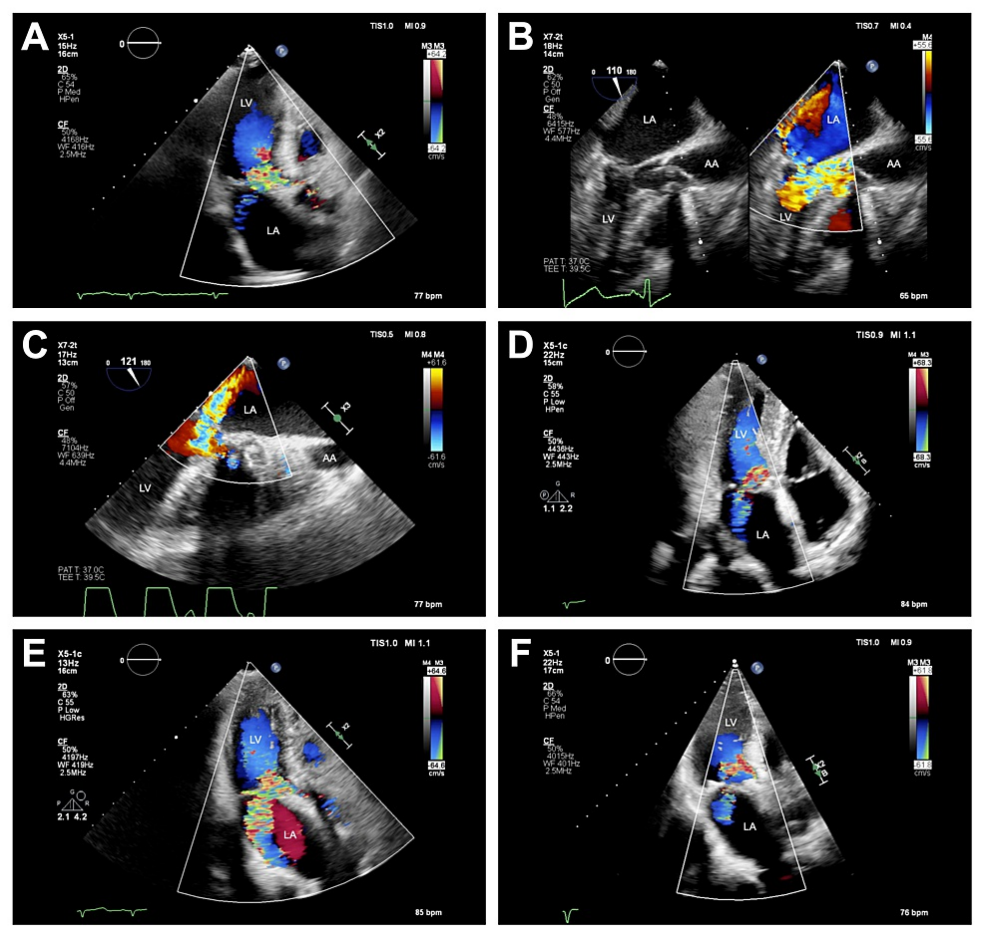
Echocardiography (A) A color Doppler image obtained in an apical long-axis view of preoperative transthoracic echocardiography shows mitral regurgitation secondary to systolic anterior motion of the mitral valve. (B) An image obtained in a mid-esophageal long-axis view using color compare imaging of intraoperative transesophageal echocardiography before implantation of a transcatheter aortic valve shows mitral regurgitation with an eccentric jet directed posteriorly to the atrial roof combined with systolic anterior motion of the mitral valve. A B-mode image is shown on the left and a color Doppler is shown on the right. (C) A color Doppler image obtained in a mid-esophageal long-axis view of intraoperative transesophageal echocardiography immediately after implantation of a transcatheter aortic valve shows apparent exacerbation of mitral regurgitation with a centrally directed jet. (D) A color Doppler image obtained in an apical long-axis view of transthoracic echocardiography after admission to the intensive care unit shows mitral regurgitation with vena contracta width of 0.46 cm and jet area to left atrial area ratio of 29%. (E) A color Doppler image obtained in an apical long-axis view of transthoracic echocardiography after the termination of the administration of landiolol and phenylephrine on postoperative day 1 shows exacerbation of mitral regurgitation with vena contracta width of 0.60 cm and jet area to left atrium area ratio of 33%. (F) A color Doppler image obtained in an apical long-axis view of transthoracic echocardiography after re-administration of landiolol and cibenzoline on postoperative day 3 shows improvement of mitral regurgitation with vena contracta width of 0.48 cm and jet area to left atrium area ratio of 27%. AA, ascending aorta; LA, left atrium; LV, left ventricle

An 88-year-old woman (height, 143 cm; weight, 41.1 kg) presenting with New York Heart Association class IV heart failure was scheduled to undergo TAVI for severe aortic stenosis. She had a history of hypertension and dyslipidemia. Preoperative laboratory investigations showed normal liver and renal functions and an elevated serum brain natriuretic peptide level of 427 pg/ml. A chest radiograph showed increased pulmonary vascular markings and a cardiothoracic ratio of 0.58. An electrocardiogram showed sinus rhythm at 77 beats/min and negative T-wave in leads V1 and V2. Transthoracic echocardiography (TTE) showed LV end-diastolic diameter of 31 mm, LV end-systolic diameter of 21 mm, and LV ejection fraction (LVEF) of 60%. There was severe aortic stenosis with a mean pressure gradient across the aortic valve of 52 mmHg. TTE also showed asymmetric LV hypertrophy with upper septal predominance (thickness of the septum, 12.0 mm; thickness of the posterior wall, 8.2 mm). Mitral regurgitation (MR) secondary to SAM was observed (Figure 1A), though a quantitative assessment was not performed. The LVOT gradient was 21 mmHg.

In the operating room, anesthesia was induced with 20 mg of propofol and 0.41 μg/kg/min of remifentanil and was maintained with 3% of desflurane and 0.12 μg/kg/min of remifentanil. At the induction of anesthesia, administration of 1.02-2.03 μg/kg/min of landiolol and 0.57-0.65 μg/kg/min of phenylephrine was also started. Mean arterial blood pressure (MAP) was 65-99 mmHg following induction of anesthesia. The operation was performed through the transfemoral route under the guidance of transesophageal echocardiography (TEE). Before valve implantation, TEE revealed MR with an eccentric jet directed posteriorly to the atrial roof combined with SAM (Figure 1B). A 26-mm Evolut™ FX self-expanding valve (Medtronic, Dublin, Ireland) was deployed under MAP of 59 mmHg induced with moderate pacing at 120 beats/min. Immediately after implantation, MAP decreased to 49 mmHg. TEE showed apparent exacerbation of MR (Figure 1C). Pericardial effusion or LV asynergy was not evident. Right ventricular pacing at 80-90 beats/min increased MAP to 55-64 mmHg. After subsequent intravenous administration of 70 mg of cibenzoline, increasing the dose of landiolol to 8.11 μg/kg/min, and boluses of colloids, MAP recovered to 62-75 mmHg. The surgery was completed in 115 min, and she was transferred to the intensive care unit.

**Figure 2:**
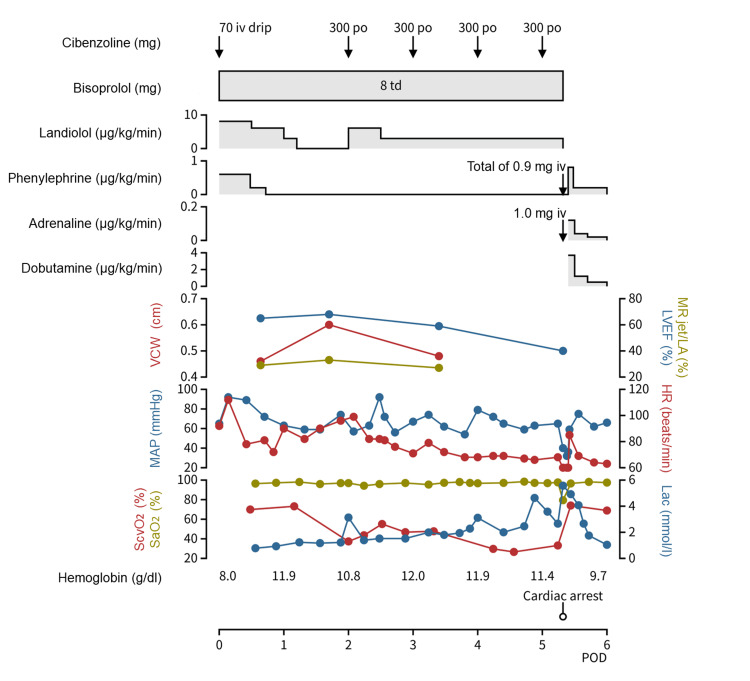
Clinical course after transcatheter aortic valve implantation HR, heart rate; iv, intravenous injection; LA, left atrium; Lac, arterial lactate level; LVEF, left ventricular ejection fraction; MAP, mean arterial blood pressure; MR, mitral regurgitation; MR jet/LA, mitral regurgitation jet area to LA area ratio; po, per os; POD, postoperative day; SaO_2_, arterial oxygen saturation; ScvO_2_, central venous oxygen saturation; td, transdermal; VCW, vena contracta width of mitral regurgitation

Her postoperative course is shown in Figure 2. When admitted to the intensive care unit, medical management consisted of 8.11 μg/kg/min of landiolol, 8 mg of a bisoprolol transdermal patch, 0.65 μg/kg/min of phenylephrine, and right ventricular pacing at 90 beats/min. TTE indicated moderate MR with vena contracta width (VCW) of 0.46 cm, jet area to left atrial (LA) area ratio of 29% (Figure 1D), and LVOT gradient of 16 mmHg. A peak jet velocity across the transcatheter heart valve was 2.1 m/s and LVEF was 65%. MAP and heart rate (HR) were 65-92 mmHg and 72-112 beats/min, respectively. Right ventricular pacing was discontinued three hours after admission to the intensive care unit because the patient's hemodynamics became more stable without pacing. On postoperative day (POD) 1, she was extubated. Landiolol and phenylephrine were tapered off and administration was terminated under hemodynamic stability with MAP of 57-77 mmHg and HR of 82-97 beats/min. However, TTE showed exacerbation of MR with VCW of 0.60 cm and jet area to LA area ratio of 33% (Figure 1E). LVOT gradient was unmeasurable and LVEF was 68%. A peak jet velocity across the transcatheter heart valve was 2.1 m/s. On POD 2, landiolol was re-administered at 3.04-6.08 μg/kg/min, and oral administration of 300 mg of cibenzoline was started. On POD 3, TTE revealed improvement of MR with VCW of 0.48 cm and jet area to LA area ratio of 27% (Figure 1F). LVOT gradient was not performed and LVEF was 59%. A peak jet velocity across the transcatheter heart valve was 2.0 m/s. MAP and HR were 54-74 mmHg and 68-79 beats/min, respectively. Hemoglobin level, arterial oxygen saturation, arterial lactate level, and central venous oxygen saturation (ScvO_2_) measured by blood gas analysis are summarized in Figure 2. After POD 3, arterial lactate level gradually increased and ScvO_2_ gradually decreased. On POD 5, she suddenly went into pulseless electrical activity. Cardiopulmonary resuscitation was performed for three minutes, resulting in the return of spontaneous circulation. Hypotension with MAP of 32-59 mmHg persisted after resuscitation. Bolus administration of 0.9 mg of phenylephrine and 0.81 μg/kg/min of phenylephrine infusion were performed; however, hypotension was refractory. After TTE demonstrated visually estimated LVEF of 40%, administration of 0.12 μg/kg/min of adrenaline and 3.65 μg/kg/min of dobutamine was started and landiolol, bisoprolol transdermal patch, and cibenzoline were all discontinued. She then presented hemodynamic stability with MAP of 61-75 mmHg. Thereafter, phenylephrine, adrenaline, and dobutamine were tapered off and administration was terminated. She was discharged to the general medical ward and oral bisoprolol of 0.625 mg was started on POD 11. On POD 14, TTE showed residual SAM with LVOT gradient of 30 mmHg, MR with VCW of 0.53 cm and jet area to LA area ratio of 23%, and normal LV systolic function with LVEF of 63%. A peak jet velocity across the transcatheter heart valve was 2.3 m/s and valve distortion was not shown. The dose of oral bisoprolol was increased to 1.25 mg on POD 15. Her hemodynamics remained stable, and she was discharged to home on POD 40 without sequelae. TTE on POD 108 showed no apparent exacerbation of MR, although quantitative evaluation of MR was difficult.

## Discussion

It was previously reported that the risk factors for SAM and LVOT obstruction after TAVI include female gender, high resting gradient, Brockenbrough-Braunwald-Morrow sign, asymmetric septal hypertrophy, and small LV cavity [1]. Our patient was deemed to be at high risk for exacerbation of MR and development of LVOT obstruction following TAVI. The effectiveness of alcohol septal ablation and transcatheter mitral valve repair as pre-emptive treatment for LVOT obstruction has been implied, but the decision to perform these interventions remains difficult [1]. Our experience was limited and thus we did not conduct the pre-emptive treatment. Instead, prophylactic medical management was performed before valve implantation. However, MR became worse immediately after TAVI and severe hypotension occurred. Since there is a correlation between the severity of MR and the magnitude of LVOT gradient [4], it was suspected that hemodynamic collapse was due to the exacerbation of MR and the development of LVOT obstruction. Intraoperative treatment with an increasing dosage of negative inotropes and fluid infusion led to recovery from hemodynamic collapse. In the postoperative period, the severity of MR changed in response to the intensity of treatment focused on negative inotropes. Collectively, the results suggested that our medical management was effective for SAM and LVOT obstruction.

Nevertheless, the patient went into cardiac arrest. Although the definitive cause of pulseless electrical activity was unclear, the postoperative trends of arterial lactate level and ScvO_2_ indicated a progressive imbalance between oxygen supply and demand. Since there was no progression of anemia or respiratory failure, arterial oxygen content was maintained. Therefore, the progressive oxygen supply-demand imbalance might have been induced by a sustained reduction in cardiac output. A depressed LVEF was verified after resuscitation, and this finding was consistent with decreased cardiac output. Importantly, after resuscitation, positive inotropes contributed to the improvement of hemodynamics and oxygen supply-demand imbalance paradoxically even though the treatment may worsen MR and LVOT obstruction [1-3]. Taken together, the postoperative course suggests that negative inotropic effects for the treatment of SAM and LVOT obstruction were excessive, possibly resulting in reduced cardiac output and, eventually, pulseless electrical activity. If more frequent echocardiographic assessment and/or accurate continuous cardiac output monitoring had been performed, the dose of negative inotropes could have been changed earlier and cardiac arrest could have been prevented.

In this case, negative inotropes included landiolol, bisoprolol transdermal patch, and cibenzoline. A previous study showed that landiolol at a maintenance dose of 10-40 μg/kg/min can increase blood pressure in patients with SAM and LVOT obstruction [5]. There have been two reports of cardiac arrest possibly caused by landiolol [6,7]. Landiolol was used at 3-10 μg/kg/min in those studies and the dosage was equivalent to that in our case. However, sinus arrest or asystole occurred in those cases, and the mechanism of cardiac arrest seems to be different from that in our case. Hepatic impairment increases the blood concentration of landiolol [8], but our patient showed normal liver function. An 8 mg bisoprolol transdermal patch can reduce the intraventricular gradient [9]. Although the effect on blood pressure may be inconstant [9], there have been no reports of cardiac arrest due to a bisoprolol transdermal patch. Cibenzoline can also be effective in decreasing the LVOT gradient [10]. However, there have been reports of cardiac arrest due to an overdose of cibenzoline [11,12]. In patients with renal impairment and in the elderly, renal clearance of cibenzoline decreases, and elimination half-life increases [13]. The patient had normal renal function but was elderly. Therefore, plasma concentrations of cibenzoline possibly have increased, resulting in cardiac arrest. A previous report indicated that the combined use of beta blockers and sodium channel blockers can enhance the effects of both medications and cause fatal hemodynamic depression [14]. In our patient, the use of multiple negative inotropes might also have led to cardiac arrest.

## Conclusions

We experienced a case of cardiac arrest possibly due to excessive negative inotropic effects even though the effects contributed to the improvement of LVOT obstruction following TAVI. Anesthesiologists and intensivists should recognize the risk of cardiac arrest induced by negative inotropic effects and use negative inotropes with rigorous hemodynamic monitoring, even when LVOT obstruction after TAVI is treated effectively.
